# Drift drives the evolution of chromosome number I: The impact of trait transitions on genome evolution in Coleoptera

**DOI:** 10.1093/jhered/esae001

**Published:** 2024-01-05

**Authors:** Heath Blackmon, Michelle M Jonika, James M Alfieri, Leen Fardoun, Jeffery P Demuth

**Affiliations:** Department of Biology, Texas A&M University, College Station, TX, United States; Interdisciplinary Program in Genetics and Genomics, Texas A&M University, College Station, TX, United States; Interdisciplinary Program in Ecology and Evolutionary Biology, Texas A&M University, College Station, TX, United States; Department of Biology, Texas A&M University, College Station, TX, United States; Interdisciplinary Program in Genetics and Genomics, Texas A&M University, College Station, TX, United States; Department of Biology, Texas A&M University, College Station, TX, United States; Interdisciplinary Program in Ecology and Evolutionary Biology, Texas A&M University, College Station, TX, United States; Department of Biology, Texas A&M University, College Station, TX, United States; Department of Biology, University of Texas at Arlington, Arlington, TX, United States

**Keywords:** chromosomal evolution, Coleoptera, genetic drift, karyotype, speciation

## Abstract

Chromosomal mutations such as fusions and fissions are often thought to be deleterious, especially in heterozygotes (underdominant), and consequently are unlikely to become fixed. Yet, many models of chromosomal speciation ascribe an important role to chromosomal mutations. When the effective population size (*N*_e_) is small, the efficacy of selection is weakened, and the likelihood of fixing underdominant mutations by genetic drift is greater. Thus, it is possible that ecological and phenotypic transitions that modulate *N*_e_ facilitate the fixation of chromosome changes, increasing the rate of karyotype evolution. We synthesize all available chromosome number data in Coleoptera and estimate the impact of traits expected to change *N*_e_ on the rate of karyotype evolution in the family Carabidae and 12 disparate clades from across Coleoptera. Our analysis indicates that in Carabidae, wingless clades have faster rates of chromosome number increase. Additionally, our analysis indicates clades exhibiting multiple traits expected to reduce *N*_e_, including strict inbreeding, oligophagy, winglessness, and island endemism, have high rates of karyotype evolution. Our results suggest that chromosome number changes are likely fixed by genetic drift despite an initial fitness cost and that chromosomal speciation models may be important to consider in clades with very small *N*_e_.

## Introduction

For any given trait, variation within and among species is a function of mutation, drift, selection, fidelity of trait expression, and isolation among lineages. Chromosome number is perhaps one of the simplest characteristics of a genome. Despite this simplicity, the relative importance of mutation, drift, and selection in generating patterns of chromosome number evolution across the tree of life has been poorly understood. Understanding the evolutionary forces that underlie strong karyotype conservation in some groups (Boyes and Shewell 1975; White 1978) while others are more labile (Kandul et al. 2007; Carbone et al. 2014) has remained elusive despite over 50 yr of work (but see Blackmon and Demuth 2014; Ross et al. 2015; Blackmon et al. 2019; Ruckman et al. 2020; Sylvester et al. 2020).

Hypotheses that propose an important role for positive selection to change chromosome number are easily allied with arguments for the costs and benefits of recombination (Muller 1932, 1964; Fisher 1958; Hill and Robertson 1966; Nei 1967; Lewontin 1971; Felsenstein 1974). When recombination is limited to one crossover per chromosome or chromosomal arm per meiosis, selection for increased or decreased recombination is expected to propel corresponding changes in chromosome number (Otto and Payseur 2019). Selection is argued to favor increased chromosome number in social insects because having more chromosomes could decrease the average relatedness within a colony—limiting the opportunity for kin-recognition-based cheating (Sherman 1979; Templeton 1979). Increased chromosome number simultaneously allows for high genotypic diversity among sibs which has been shown to benefit colony growth, efficiency, and pathogen resistance (Tarpy 2003). However, analyses comparing solitary and social Hymenoptera show that the strength of this selection on chromosome number is likely weak (Ross et al. 2015). Positive selection may also be particularly important in decreasing the number of autosomes through selection for fusions between autosomes and sex chromosomes to resolve sexual antagonism (Charlesworth and Charlesworth 1980; Anderson et al. 2020; Sylvester et al. 2020).

Counter to the general applicability of positive selection’s role as an agent of karyotype evolution (King 1995), empirical observations suggest that most changes in karyotype are either neutral or deleterious, with many being underdominant (i.e. deleterious when heterozygous). Underdominance of karyotype changes can result from difficulties in meiosis, where mismatched chromosomal types do not segregate properly. Such underdominant mutations are only expected to fix when natural selection is overcome by random genetic drift in small populations (Wright 1941; Lande 1979, 1985). It is also important to note that in a small population, an underdominant mutation has a higher frequency when it first occurs than in a large population with the initial frequency being the reciprocal of two times the population size. This means that it can reach a higher frequency through drift more quickly, and once it is the major allele in the population, it ceases to have a negative selection coefficient. Despite the difficulties of fixing underdominant mutations, attempts to ascribe a causal role to karyotype evolution in the speciation process have been abundant (Lewis 1966; White 1978; Bickham 1979; Grant 1981; Templeton 1981; Baker and Bickham 1986; Rieseberg 2001). Some models assume karyotype changes are neutral but facilitate diverging local adaptation by sheltering some genome regions from the homogenizing effects of gene flow and recombination (Guerrero and Kirkpatrick 2014). Chromosomal changes could even function under a Bateson–Dobzhansky–Mueller incompatibility model where two isolated populations fix neutral changes that, when combined into a single genome, lead to segregation defects and function as an isolating barrier upon secondary contact (Baker and Bickham 1986).

It is important to note that most information on the fitness effects of chromosomal mutations may not be representative of the changes that produce extant diversity. For instance, many examples have relied on crosses between divergent strains assessing fitness in offspring (Ratomponirina 1988; King 1995). However, because the parental strains or species have often been isolated for millions of years, fitness effects may be compounded by genic incompatibility (Bayes and Malik 2009). Other evidence comes from induced chromosomal changes using mutagenic techniques that produce changes that may be fundamentally different from naturally occurring chromosomal mutations (Roberts 1967, 1970). By leveraging phylogenetic comparative methods to analyze extant diversity, we believe we can more directly characterize the relative importance of mutation, selection, and drift in karyotype evolution and estimate the typical fitness effect of mutations that have led to observed divergence in chromosome number.

Given that effective population size (*N*_e_) governs the efficacy of natural selection in relation to random genetic drift, one way to distinguish between these two evolutionary forces is to see whether factors associated with differences in *N*_e_ are also associated with differences in the rate of karyotype evolution and or the direction of change in chromosome number. If karyotype changes are neutral, their fixation rate should be unrelated to *N*_e_. However, if karyotype changes are deleterious, then species with small *N*_e_ should have higher rates of chromosome number evolution as more changes are fixed by drift. In contrast, if selection plays a broad role, we expect species with larger *N*_e_ to have higher rates. Ultimately rates of karyotype evolution will be determined by the interplay of mutation, selection, and drift. Some empirical examples have illustrated the importance of mutation rate and selection (Carbone et al. 2014; Ross et al. 2015). What remains unclear is whether these are the exception or the rule.

Efforts to relate *N*_e_ to karyotype evolution typically use ecological and phenotypic traits as proxies for expected differences in *N*_e_. For instance, all else being equal, winged species should have larger *N*_e_ than wingless species because flight increases dispersal distances (*N*_e_ = 4*πσ*^2^*δ*), where *σ*^2^ is a measure of dispersal distance and *δ* is the density of adults per unit area (Wright 1946). Mating system has also been used where species classified as inbreeding are assumed to have lower *N*_e_ than outbreeding species (Charlesworth 2003). Geographic distribution can be divided into continental and island endemics, with island endemics being assumed to have lower *N*_e_. Finally, the degree of diet specialization can be used with mono or oligophagous species having lower *N*_e_ than polyphagous species (Li et al. 2014).

The distribution of these traits has then been compared with the rate of karyotype change as estimated by scaling the variance in chromosome number to a fossil date for the taxonomic group of interest (Wilson et al. 1975; Bush et al. 1977; Bengtsson 1980; Imai et al. 1983; Larson et al. 1984; Petitpierre 1987; Olmo 2005). These earlier studies suggest that taxa inferred to have highly structured populations also have faster rates of karyotype evolution. There is also a negative correlation between the estimated rate of karyotype evolution and allozyme heterozygosity levels in some cases (Coyne 1984). These studies have generally supported a hypothesis of random genetic drift in small populations driving increased rates of chromosome change. However, previous work has been limited by not incorporating phylogenies or evolutionary models for chromosome change and using comparisons between highly divergent clades where the underlying mutation rates and accuracy of fossil ages may differ dramatically (i.e. across all vertebrates).

The present study expands previous efforts to understand karyotype evolution in two ways. First, we incorporate ecological and phenotypic proxies for *N*_e_ with a comprehensive database of Coleoptera karyotypes (Blackmon and Demuth 2014). Second, we employ a modern statistical phylogenetic framework to model the relationship between *N*_e_ and chromosome evolution (Blackmon et al. 2019). Coleoptera is an excellent group to study the effect of *N*_e_ on chromosome evolution because they exhibit variation in all proxies for *N*_e_ (Crowson 1981), have a good phylogenetic scaffold, and contain an extensive database of karyotypes (4,957 species). We show that lineages with multiple traits associated with reduced *N*_e_ (loss of wings, inbreeding, island endemism, restricted feeding) also have faster rates of karyotype evolution. Our findings suggest that random genetic drift is the predominant driver of fast karyotype evolution, as predicted if changes in chromosome number are typically underdominant or mildly deleterious.

## Methods

### Data collection

We compiled all available karyotypes from the Coleoptera Karyotype Database (www.karyotype.org) (Blackmon and Demuth 2015). Coleoptera karyotypes rarely include banding data and are typically reported as the meioformula, consisting of the number of autosomes plus the sex chromosome complement of the male. For this reason, we use male haploid autosome count as a surrogate for karyotype, and for the remainder of the paper, refer to this as the chromosome number. In 19 cases where multiple values were reported for a species, each value was retained, and these distributions were sampled in downstream analyses to account for tip state uncertainty.

Lack of overlap in the available data for karyotype, phylogeny, and *N*_e_-related traits resulted in our analysis being subdivided into a family-level analysis of Carabidae using the presence or absence of wings and a sparser but more phylogenetically diverse analysis of 12 clades using multiple *N*_e_-influencing traits. Data for the presence of wings in Carabidae were taken from a previous compilation of natural history data (Larochelle and Larivière 2003). Species reported as being polymorphic for wings were scored as having an equal probability of being either winged or wingless. To maximize the amount of data that could be analyzed, if wing data were not present for a species in the karyotype and phylogenetic datasets, but other species in the genus were reported, the species was assigned a probability reflecting available data for the genus. For instance, in the genus *Calathus*, 64% of species were reported as wingless; therefore, all *Calathus* species not in the trait dataset were also assigned a 64% probability of being wingless and 36% probability of being winged.

For each of the 12 clades included in our clade-level rate estimates, we performed literature searches to score the included species for the following traits: winged vs. wingless, inbreeding vs. outbreeding, island vs. continental distributions, and mono/oligophagy vs. polyphagy. If any species included in a clade dataset had a low *N*_e_ version of a trait, the clade was scored as having this trait. This process led to categorizing clades into those with 0 to 2 low *N*_e_ traits. We use this number of low *N*_e_ traits occurring in a clade to classify them into high, medium, or low expected *N*_e_ classes (Table 1). To account for phylogenetic history in our comparative analysis, we used 100 trees from the posterior distribution produced in an earlier study (Blackmon and Demuth 2014). Briefly, these trees are based on an analysis of seven genes (16s, 18s, 28s, COI, elongation factor 1, arginine kinase, and wingless) across 1,042 taxa in BEAST (v1.7.5; Drummond and Rambaut 2007; Suchard and Rambaut 2009). We assumed a lognormal relaxed clock and used normal distributions to place priors on the age of seven nodes where ages were based on previous estimates (McKenna and Farrell 2009).

**Table 1.** Distribution of traits likely to impact effective population size.

	Clade (*N*)	Breeding	Feeding	Distribution	Wings	Expected *N*_e_
Adephaga	*Bembidion* (40)	+	+	+	+	High
	*Calathus* (15)	+	+	−	−	Low
	*Cicindelidae* (27)	+	+	+	+	High
	*Harpalus* (14)	+	+	+	+	High
	*Pterostichus* (15)	+	+	+	−	Medium
Polyphaga	*Chrysolina* (25)	+	−	+	−	Low
	*Cyrtonus* (13)	+	−	+	−	Low
	*Dendroctonus* (13)	−	−	+	+	Low
	*Diabrotica* (12)	+	+	+	+	High
	*Ips* (26)	+	−	+	+	Medium
	*Pimelia* (29)	+	+	+	−	Medium
	*Timarcha* (30)	+	−	+	−	Low

Traits were scored based only on species included in the analysis, and the majority state is reported. Expected *N*_e_ is categorized by the number of *N*_e_-impacting traits in a clade. High, medium, and low *N*_e_ categories were assigned if a clade had zero, one, or two *N*_e_-reducing traits, respectively. A (+) indicates the high *N*_e_ version of a trait, and a (−) indicates the low *N*_e_ version of a trait. After each clade name, we include the number of species in the analysis.

### Inferring rates of chromosome evolution

**Fig. 1.**
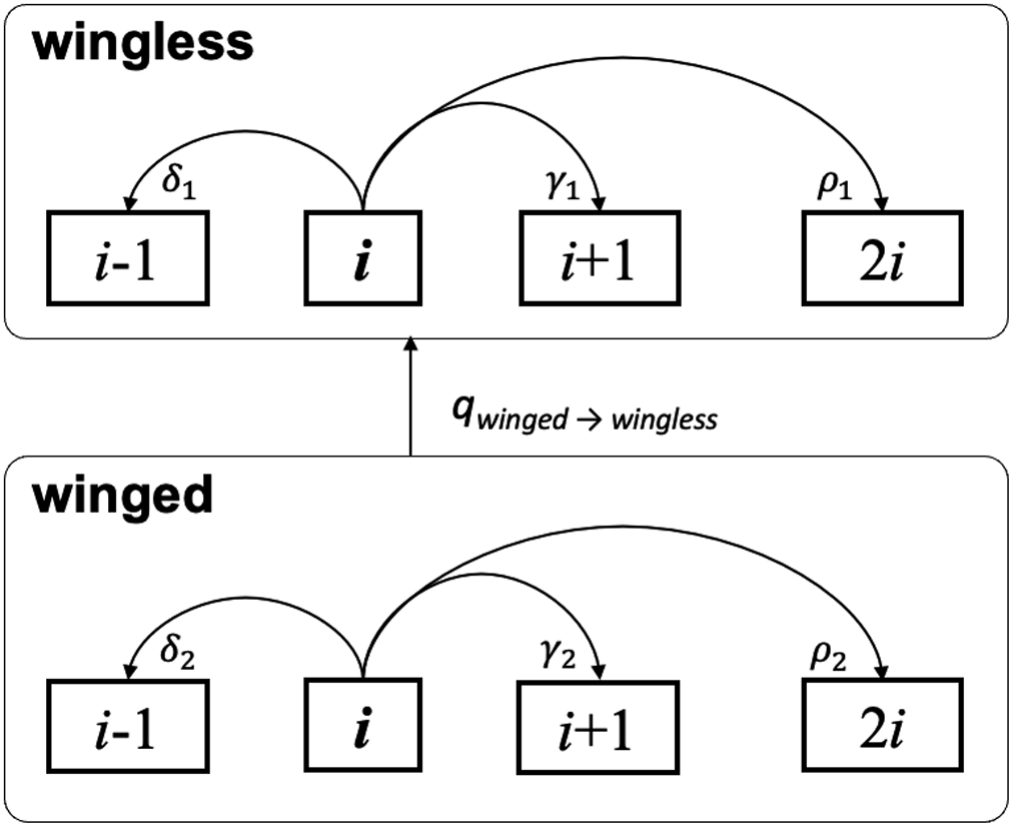
Model for the evolution of chromosome number in wingless and winged clades. At an instance in time, a lineage will have *i* chromosomes and be in a wingless or winged state. A lineage can make three transitions in chromosome number: fusion (*δ*), fission (*γ*), whole-genome duplication (*ρ*), and if currently winged, can transition to wingless (*q*_winged→wingless_). Rates of fusion, fission, and whole-genome duplication are allowed to differ between winged and wingless lineages.

We used the R package ChromePlus to construct a Markov model of chromosome evolution (Fig. 1) that allows transitions in a binary character (i.e. wing presence or absence) and three mechanisms of chromosome number change: fusions (*δ*), fissions (*γ*), and whole-genome duplication (*ρ*) that can vary depending on the state of the binary character (Blackmon et al. 2019, 2023; Ruckman et al. 2020). Because the evidence for whole-genome duplication is limited, we also fit a model where the rate of whole-genome duplication was set to zero (Li et al. 2018). This model was fit in a Bayesian framework using the R package diversitree on each of the 100 phylogenies from the posterior distribution of phylogenies (FitzJohn 2012). Each Markov Chain Monte Carlo (MCMC) run was initialized with parameter values drawn from a uniform distribution from 0 to 10. We applied a broad exponential prior with a shape parameter of 2 to avoid sampling unrealistically high rates. We repeated the MCMC on all 100 trees for 200 generations. All runs converged within the first 25 steps. However, we conservatively discarded the first 100 generations as burnin for each MCMC run. Posterior distributions for rate parameters were compared with the prior distribution to ensure that the prior was not unduly influencing the inference of the posterior distribution. In this model, all rates reported are lambda parameters for exponential distributions that describe the expected waiting time for a transition to occur. To allow for easy interpretation of rates during the fitting process, we scaled trees to unit length; however, all rates reported here have been rescaled to convert to units of millions of years.

To determine whether wingless clades have higher fusion, fission, and whole-genome duplication rates, we report our results in terms of a mean rate difference statistic, Δ*R*_*x*_, where *x* is the model parameter of interest. For instance, for the rate of fusions (*δ*) for each post-burnin sample, we calculated Δ*R*_*δ*_ as:


$$\begin{array}{l} \;\Delta \;R_{\delta }=\delta_{Wingless}-\delta_{\;\;\;Winged} \end{array}$$

We then examine the 95% credible interval of Δ*R*_*δ*_. In cases where the credible interval of Δ*R*_*δ*_ is above zero, there is strong support for higher fusion rates in wingless lineages. If the credible interval of Δ*R*_*δ*_ is below zero, there is strong support for higher fusion rates in winged lineages. In cases where the credible interval of Δ*R*_*δ*_ overlaps zero, our interpretation is that there is limited or no support for differences in the rate of fusion between wingless or winged lineages.

For our clade-level analyses, we used two simplified chromosome evolution models, one with only fissions (*γ*) and fusions (*δ*) and one with fissions (*γ*), fusions (*δ*), and whole-genome duplication (*ρ*). These models were fit separately for each clade in our analysis. Model fitting and MCMC approaches were identical to those for the wingless analysis. In all analyses, if a tip had multiple chromosome numbers reported, we randomly sampled among possible values each time the model was fit on one of the 100 trees from the posterior distribution. Using this approach, we incorporate both uncertainties in phylogeny and tip states. All analyses were completed in R version 4.1.1 (R Core Team 2021). Since previous work has shown that different rates of chromosome evolution may occur in the two major suborders of beetles (Blackmon and Demuth 2014), we analyzed the clades in each suborder separately.

### Scaled variance estimates

Since the lack of overlap between species trait data and existing phylogenetic information causes a considerable reduction in the number of data points in our analysis, we also investigated whether estimating the rate of karyotype evolution without incorporating phylogenies (as many past studies have done) is consistent with the phylogenetic model-based approach above. We calculated time-scaled coefficients of variation by first locating the oldest available fossil record for each clade of interest in the Paleobiology Database (PaleoDB) (http://paleodb.org). We then used the fossil ages to scale the coefficients of variation for chromosome number in each taxon. To assess consistency between these “scaled variance” estimates and the phylogenetic model-based rate estimates, we calculated a mean rate of chromosome evolution in each taxon by taking the mean of the fission, fusion, and whole-genome duplication rates in each generation of the MCMC and then taking the means of these to arrive at a single value for each taxon. We then used a nonparametric correlation analysis (Kendall’s *τ*). All tests were considered significant at *P*-value <0.05. Scripts for all analyses are available in a GitHub repository (https://github.com/coleoguy/coleochroms).

## Results

### Data collection

**Fig. 2.**
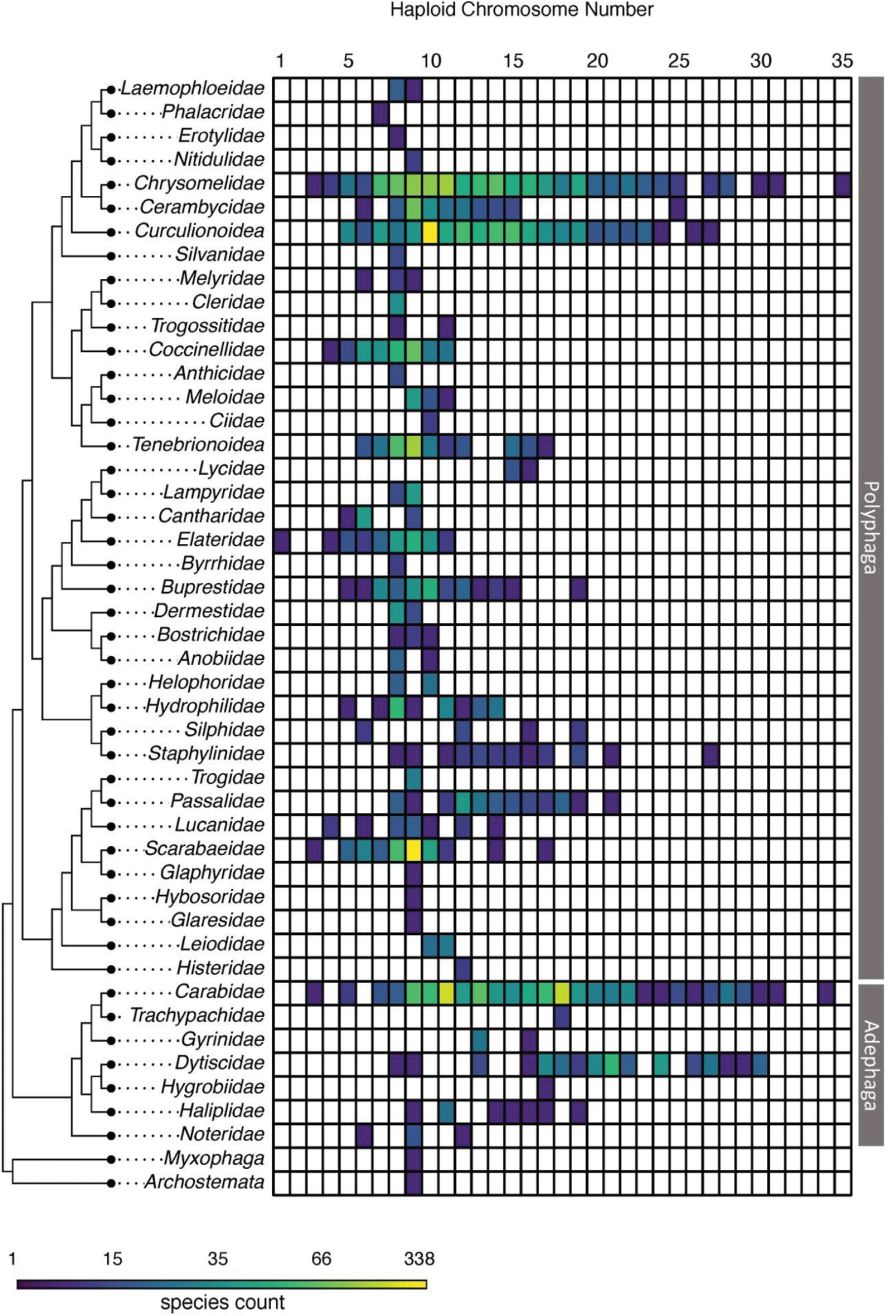
Haploid chromosome counts in Coleoptera. Each row represents a family of beetles, and each column indicates a male haploid autosome count. The shading of each cell represents the number of records with a given haploid count with colors on a log scale.

We downloaded 4,957 records from the Coleoptera Karyotype Database (karyotype.org). Our karyotype dataset included data for all four extant suborders of Coleoptera. Two of these suborders (Myxophaga and Archostemata) are represented by only one and two karyotypes; therefore, we focused our analysis on the larger suborders of Adephaga and Polyphaga. These two suborders accounted for 1,285 and 3,669 karyotypes, respectively. In Adephaga, the number of autosomes ranged from 3 to 34 (mean = 15.57 ± 0.14), while in Polyphaga, the range was from 1 to 35 (mean = 10.63 ± 0.06). Polyphaga exhibits a single mode of nine autosomes, accounting for 952 species or 29% of all Polyphaga records. Conversely, Adephaga is bimodal, with concentrations at 11 and 18 autosomes accounting for 276 and 242 species or 23% and 20%, respectively (Fig. 2).

### Phylogenetic model-based rate estimates

**Fig. 3.**
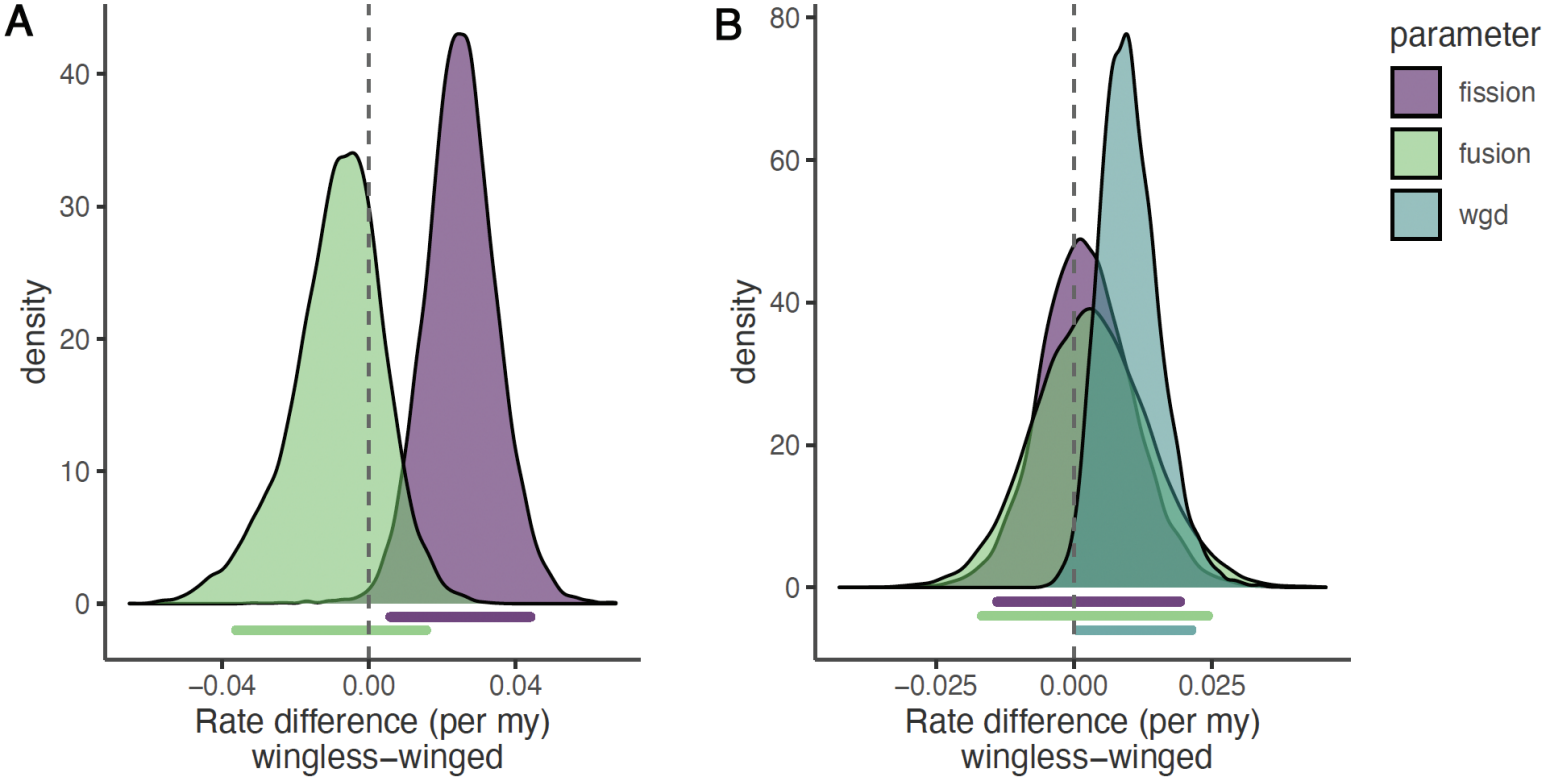
Differences in rates of chromosome evolution in winged and wingless Carabidae. In each plot, we show the distribution of the differences in rates of wingless minus winged clades. The credible interval for each statistic is shown below the distribution. A) simple model with just fissions and fusions B) complex model with fission, fusions, and whole-genome duplication.

Karyotypes for 1,065 Carabidae species were available, and 136 of these were used in our comparative analysis because they were included in our phylogenetic tree and had data available on flight ability. These data were used to fit the two models (with and without whole-genome duplication). We evaluated results for more complex models that would allow wing gain and loss, but this has little support biologically and produced results that were qualitatively the same. All results below are for a model where wings can be lost but not regained. In the simplest model with only fusions and fissions, we estimate a mean Δ*R*_fusion_ of −0.009 and a mean Δ*R*_fission_ of 0.025. For Δ*R*_fusion_, the credible interval spanned zero. In contrast, the credible interval for Δ*R*_fission_ was entirely positive (0.005 to 0.044; Fig. 3A). In our more complex model that includes whole-genome duplication, we estimate a mean Δ*R*_fusion_ of 0.003 and a mean Δ*R*_fission_ of 0.002. For both Δ*R*_fusion_ and Δ*R*_fission_, the credible interval spanned zero. The mean estimate for Δ*R*_wgd_ was 0.010, and the low end of the credible interval was just above zero (2.3 × 10^−5^; Fig. 3B). In examining the posterior distribution, we found that just 1.3% of the 10,000 samples from the posterior distribution sampled regions with a negative Δ*R*_wgd_. Taken together, these results suggest that wingless Carabidae have higher rates of increases in chromosome number (Fig. 3).

For the analysis of clade level, we scored each clade for the presence or absence of traits thought to reduce *N*_e_. This allowed us to assign each clade to a class based on expected *N*_e_. Four clades possess no *N*_e_-reducing traits, and we classify these as the high *N*_e_ class. Three possess only one of these traits and form the medium *N*_e_ class. Five clades (*Calathus*, *Chrysolina*, *Cytronus*, *Dendroctonus*, and *Timarcha*) possess two of the *N*_e_-reducing traits, which form the low *N*_e_ class (Table 1).

For our clade-level analysis, we selected all clades where the phylogeny and karyotype data overlap included at least 12 species. This led to 12 clade-level analyses (five in the suborder Adephaga and seven in the suborder Polyphaga). One of these was the genus *Cicindela* (tiger beetles). More broadly, *Cicindela* and tiger beetles have been the focus of intense taxonomic studies and changes (Gough et al. 2019, 2020). In light of the taxonomic instability, we chose to include all species in the family Cicindelidae in our analysis of this clade (Duran and Gough 2020).

**Fig. 4.**
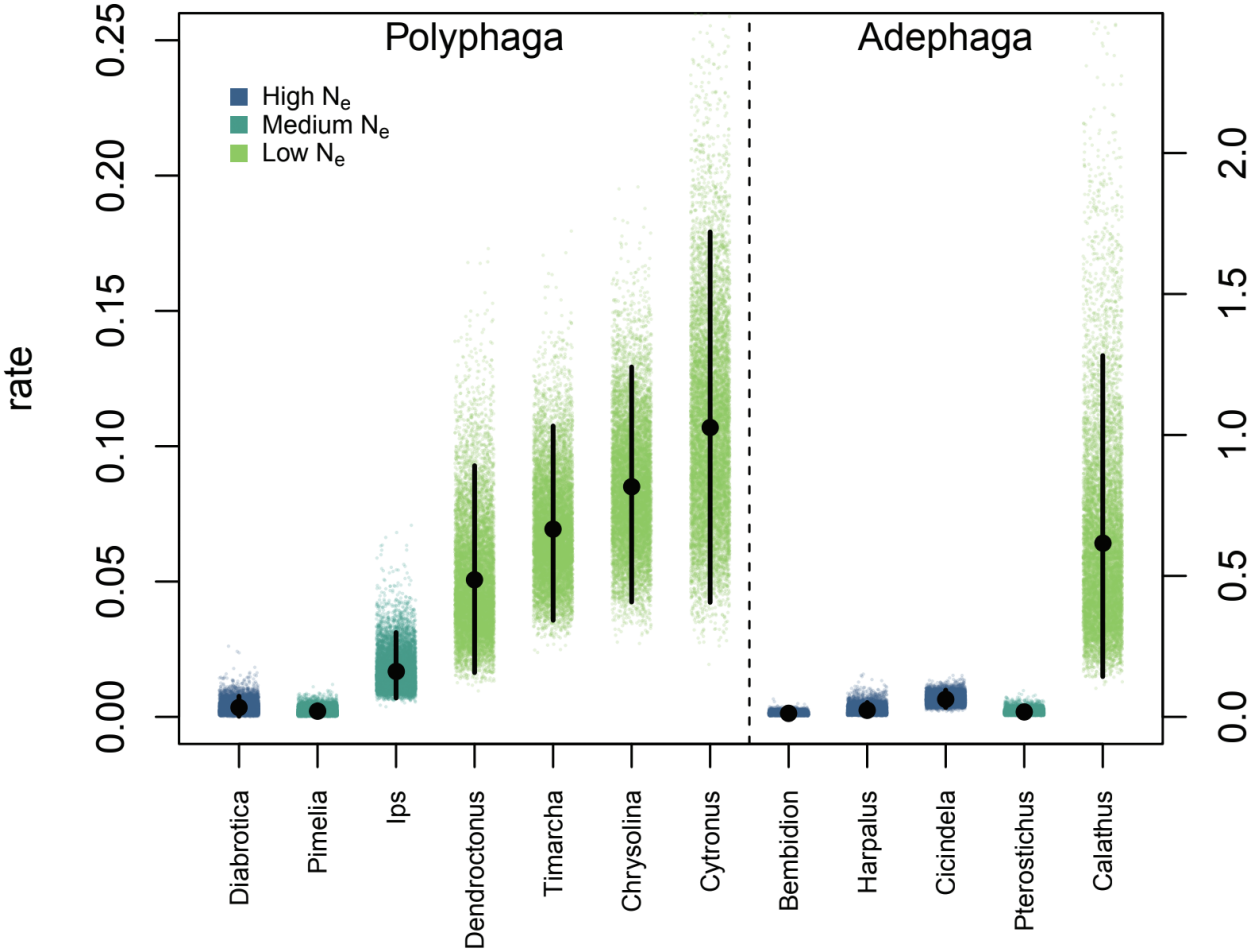
Mean rates of chromosome evolution in Coleoptera clades. For each clade, we plot 10,000 samples from the posterior distribution of mean rate estimates from each generation of the MCMC. The mean of the posterior distribution is indicated with a black circle, and the credible interval is indicated with a vertical black line. Rates for Polyphaga are plotted on the scale to the left, while rates for Adephaga are plotted on the scale to the right; the dashed line separates those clades plotted against different axes. The color of points indicates *N*_e_ class.

In our analysis of Polyphaga clades, we found that all four clades that we placed in the low *N*_e_ size class had a higher mean rate than the three clades placed in the medium and high *N*_e_ classes. Three low *N*_e_ clades had credible intervals that did not overlap with the medium and high *N*_e_ classes (Fig. 4). In our analysis of Adephaga clades, we found a similar pattern. The one genus (*Calathus*) that we scored as being in the low *N*_e_ class had a higher mean rate and credible interval than all medium and high *N*_e_ clades. We interpret this as strong evidence that traits expected to have low *N*_e_ are associated with increased rates of chromosome number evolution.

### Estimates based on scaled variance

For each of the 12 clades included in our rate estimate inference, we searched PaleoDB for fossil records. Nine of our target clades had fossil data available; if multiple dates were available, we recorded the age of the oldest available record. One taxon, *Ips*, was included based on a fossil that reflects the family rather than the genus. For this reason, we substituted an age for this genus that reflects the median age estimate based on a time tree analysis that used several fossils as priors on nodes (Gohli et al. 2017). These ages ranged from a low of 7.24 million yr for *Chrysolina* to 150.8 million yr for Cicindelidae (Table 2). We calculated the scaled variance of chromosome number for each of the nine clades by dividing the coefficient of variation for chromosome number by the fossil-based age estimates (Table 2). These scaled variances ranged from 0.0005 in *Diabrotica* to 0.32 in *Dendroctonus.* Notably, we find no correlation between the scaled variance-based estimates of karyotype evolution and the phylogenetic model-based rate estimates for the nine clades with overlapping analyses (*τ* = 0.11, *P*-value = 0.76).

**Table 2.** Clade-level phylogenetic and scaled variance-based estimates of the rate of chromosome evolution.

Clade	NMR^a^	MR^b^	NSCV^c^	CV^d^	Age	SCV^e^	Fossil reference
*Bembidion*	40	0.096	213	0.046	48.6	0.096	Arillo and Ortuño (1997)
*Calathus*	16	0.375	19	0.107	28.4	0.375	Martynov (1929)
*Chrysolina*	26	4.215	59	0.305	7.24	4.125	Hopkins et al. (1971)
*Cicindelidae*	27	0.147	105	0.222	150.8	0.147	Handlirsch (1906)
*Dendroctonus*	13	0.837	20	0.386	46.2	0.837	Labandeira et al. (2001)
*Diabrotica*	12	0.050	36	0.018	37.2	0.050	Wickham (1914)
*Harpalus*	14	0.082	29	0.054	65.5	0.082	Birket-Smith (1977)
*Ips*	26	0.507	35	0.180	40.0	0.450	Gohli et al. (2017)
*Pterostichus*	15	0.259	49	0.096	37.2	0.259	Wickham (1914)

For ease of presentation model-based rates and scaled variances are multiplied by 100.

^a^Number of taxa used in phylogenetic model-based estimate of rates.

^b^The mean rate of chromosome change (phylogenetic approach).

^c^Number of taxa used in calculation of scaled coefficient of variance (SCV).

^d^Coefficient of variation.

^e^Scaled SCV=CV/age.

## Discussion

Our analyses clearly show that traits that are expected to reduce *N*_e_ are associated with increased karyotype evolution rates, and this pattern holds independently of potential differences in mutation rate between lineages. The most likely explanation for this pattern is that chromosome number changes are predominantly deleterious while segregating and become fixed by random genetic drift.

In our analysis of Carabidae, we fit two models. The first was a complex model that allowed fusion, fission, and whole-genome duplication. Under this model, only the rate of whole-genome duplication was higher in wingless lineages than in winged lineages. However, when we fit a simpler model with fusions and fissions we find that the rate of fission is higher in wingless lineages, but the rate of fusion was similar in all lineages. So in this case regardless of the model increases in chromosome number occur more frequently in lineages that have lost wings.

In our analysis of the Polyphaga and Adephaga clades, the difference between the low *N*_e_ clades and the medium and high *N*_e_ clades is striking. In Polyphaga, the mean rate of fusions in low *N*_e_ clades ranges from 0.05 to 0.11. In contrast in medium and high *N*_e_ clades mean rates were all below 0.025. In Adephaga, only one clade is classified as low *N*_e_ (*Calathus*). However, *Calathus* exhibits strikingly high rates compared with the other four clades. All rates for *Calathus* were more than ten times higher than the average of the other Adephaga clades.

A few notable examples serve to highlight this overall pattern in beetles. First, within the Polyphaga family Curculionidae, our study includes the closely related scolytid genera *Ips* and *Dendroctonus*. Our estimate for the mean rate of karyotype evolution in *Dendroctonus* 0.025 was three times higher than the mean rate in *Ips* 0.0083. This marked difference matches expectations based on breeding behavior. *Dendroctonus* is an inbreeding genus producing biased sex ratios and practicing predispersal sibmating (Grégoire 1988). Meanwhile, *Ips* is an outbreeding genus where both males and females disperse, and neither sibmating nor biased sex ratios have been documented (Kirkendall 1993). These characteristics should lead to smaller *N*_e_ in *Dendroctonus* and allow changes in karyotype to be fixed more easily even if they are underdominant, as theory predicts. Second, the three highest rates of karyotype evolution in Polyphaga were observed in the Chrysomelidae genera *Cyrtonus*, *Chrysolina*, and *Timarcha*, all of which have wingless, oligophagous species. In all three genera, the mean of the posterior distribution for the rates of karyotype evolution was higher than in *Diabrotica*, a chrysomelid genus lacking any of the small *N*_e_ traits.

One potentially confounding factor in our analysis is the inclusion of pest species that experience periodic outbreaks that can lead to orders of magnitude increase in census population size (Kunegel-Lion and Lewis 2020). If outbreaks were frequent or lasted many generations this could lead to species with high effective population size despite exhibiting traits that we hypothesize to be associated with low effective population size. However, studies in locust suggest that effective population size is often quite similar in comparisons between populations experiencing outbreaks and those that are not (Chapuis et al. 2009). Outbreaking populations do typically exhibit higher levels of gene flow which can homogenize long-term effective population size across a species (Chapuis et al. 2008, 2009). The degree to which this result would be similar for strict sibmating species is unclear.

Finally, the genus *Calathus* provides the best example of the compound effects of phenotype and ecological history on the tempo of karyotype evolution. First, many species in *Calathus* are wingless and thus may be characterized by populations composed of small demes where fixation of karyotype changes should be more likely. However, the exceptionally high rate estimate in this genus is likely driven by just two taxa in our dataset. *Calathus abaxoides* and *C. ascendens*, these species have the highest and lowest chromosome numbers in the genus. Interestingly, *C. abaxoides* and *C. ascendens* are both endemic, wingless, *Calathus* species on the Canary archipelago. Both species occur on the island of Tenerife, which the genus has colonized in the last 12 million yr (Emerson et al. 1999). These species likely experienced an initial population bottleneck during colonization, and the continued restriction to an island has led to a sustained lower *N*_e_ than species with continental distributions. While 17 to 19 autosomes are common for most species in this genus, *C. abaxoides* has increased to 27 autosomes, while *C. ascendens* has decreased to 10 autosomes. The observation that both the lowest and highest chromosome number are the product of a single recently colonized island further suggests that drift in small populations is responsible for rapid karyotype evolution.

### The role of mutation

Little is known about mutation rate variation in beetles; however, we recently hypothesized that differences in the mechanisms of meiosis might provide a mutational basis for differences in the rate of sex chromosome turnover between the two main beetle suborders, Polyphaga and Adephaga (Blackmon and Demuth 2014). The present analysis demonstrates that the two suborders also have very different overall rates of chromosome evolution (note the difference in scales between the left and right vertical axes of Fig. 4) that are consistent with our findings on sex chromosome rates. It is noteworthy, however, that despite this difference in the “background” rate of karyotype evolution, the pattern where small *N*_e_ is associated with relatively rapid karyotype evolution holds within both suborders. Thus, while mutation rate may be a major factor driving the baseline rate of karyotype evolution, our analysis suggests that within a given mutational context, most changes are at least mildly deleterious and become fixed by random genetic drift.

### Comparison with previous work

While our findings accord with earlier studies relating *N*_e_ to variation in chromosome number, our phylogenetic model-based rate estimates are not correlated with time-scaled variance estimates derived similarly to previous work. This inconsistency is worth noting because the scarcity of reliable phylogenies limits analyses in other groups. The lack of consistency between approaches highlights the risk inherent in ignoring the pattern of chromosome evolution over the phylogeny. Theoretically, a scaled variance method could work. However, its accuracy will be limited by the extent to which the ages estimated for the groups are accurate and correlated with the total phylogenetic branch lengths relating to the focal taxa. These requirements are unlikely to be met, particularly in groups with relatively incomplete and highly heterogeneous fossil records, such as insects. Methods not using a phylogeny will also be misled when the number of records is insufficient to capture the true variance of the groups being studied. This sampling issue is less of a problem when phylogenetic methods are used because the observed phenotypic divergence is accounted for within the context of the divergence times among the sampled species. In contrast, without a phylogeny, variance among sampled clades must be assumed to be a true measure of variance for the focal clade. The variance in chromosome number across families of Coleoptera can be partly explained by the number of records available (Pearson’s correlation coefficient between family variance and the number of records = 0.41, *P*-value = 0.008). This suggests that some families have not been sampled sufficiently to capture the true variance of extant species. Applying an evolutionary model for karyotype evolution using a time-scaled phylogeny eliminates these issues.

## Conclusion

Our results, in concert with previous work, suggest that chromosome number evolution is primarily governed by random genetic drift in small populations and that mutations that change chromosome number are deleterious (at least while segregating). Despite this finding there are almost certainly many individual cases where selection has driven a change in the karyotype (Kitano et al. 2009; Blackmon et al. 2019). However, the association we find between factors influencing *N*_e_ and evolutionary rate also puts bounds on the selection coefficient of mutations that change chromosome number and go on to be fixed, suggesting that many changes are likely to be only mildly deleterious; otherwise, reduced *N*_e_ due to ecological and phenotypic transitions in Coleoptera would not be sufficient to drive significant increases in the number of chromosome changes that are fixed by random genetic drift.

More broadly, our work suggests that when species evolve traits or inhabit locations that restrict population size, the rate of change in chromosome number often increases by orders of magnitude relative to closely related species. Increasing the fixation rate of karyotype changes makes speciation mechanisms requiring genome rearrangements more likely. This should be true for models that assume underdominance of karyotype mutations, such as those described by White (1978), and more recent models that assume karyotype changes to be neutral such as those described by Rieseberg (2001). Traditionally, chromosomal rearrangements are thought to be more likely to contribute to speciation in plants than animals, possibly due to gene expression in pollen or lack of differentiated sex chromosomes in most plants (reviewed in Rieseberg 2001). Chromosomal speciation has also been suggested to be more likely in mammals than invertebrates due to differences in meiosis (Coyne and Orr 2004). However, given that our results suggest most karyotype changes in beetles are deleterious while segregating, models that invoke karyotypic changes acting directly as reproductive barriers seem more widely plausible than they have been considered recently. Unfortunately, the coarse nature of karyotype data limits our analysis to mutations such as fusions and fissions that change the number of chromosomes. Future work incorporating genomic data would be helpful to determine whether other types of mutations, such as inversions and translocations, also reflect a similar pattern.

## Data Availability

All data and scripts necessary to replicate the analyses and figures in this manuscript are available via a GitHub repository: https://github.com/coleoguy/coleochroms.

## References

[CIT0001] Anderson NW , Hjelmen CE, Blackmon H. The probability of fusions joining sex chromosomes and autosomes. Biol Lett. 2020:16:20200648. 33232649 10.1098/rsbl.2020.0648 PMC7728677

[CIT0002] Arillo A , Ortuño VM. First records of the families Anthicidae and Chrysomelidae from the Oligocene of Izarra (Alava, Spain). Coleopt Bull. 1997:51:293–297.

[CIT0003] Baker RJ , Bickham JW. Speciation by monobrachial centric fusions. Proc Natl Acad Sci USA. 1986:83:8245–8248. 16593777 10.1073/pnas.83.21.8245 PMC386904

[CIT0004] Bayes JJ , Malik HS. Altered heterochromatin binding by a hybrid sterility protein in *Drosophila* sibling species. Science. 2009:326:1538–1541. 19933102 10.1126/science.1181756 PMC2987944

[CIT0005] Bengtsson BO. Rates of karyotype evolution in placental mammals. Hereditas. 1980:92:37–47. 6991455 10.1111/j.1601-5223.1980.tb01676.x

[CIT0006] Bickham JW. Canalisation model of chromosomal evolution. Bull Cargnegie Mus Nat Hist. 1979:13:70–84.

[CIT0007] Birket-Smith SJR. Fossil insects from Spitsbergen. København: CA Reitzels Forlag; 1977.

[CIT0008] Blackmon H , Chin M, Jonika M. coleoguy/chromePlus. chromePlus; 2023.

[CIT0009] Blackmon H , Demuth JP. Estimating tempo and mode of Y chromosome turnover: explaining Y chromosome loss with the fragile Y hypothesis. Genetics. 2014:197:561–572. 24939995 10.1534/genetics.114.164269 PMC4063915

[CIT0010] Blackmon H , Demuth JP. Coleoptera karyotype database. Coleopt Bull BioOne. 2015:69:174–175.

[CIT0011] Blackmon H , Justison J, Mayrose I, Goldberg EE. Meiotic drive shapes rates of karyotype evolution in mammals. Evolution. 2019:73:511–523. 30690715 10.1111/evo.13682 PMC6590138

[CIT0012] Boyes JW , Shewell GE. Cytotaxonomy of Calliphoridae (Diptera). Genetica. 1975:45:435–488.

[CIT0013] Bush GL , Case SM, Wilson AC, Patton JL. Rapid speciation and chromosomal evolution in mammals. Proc Natl Acad Sci USA. 1977:74:3942–3946. 269445 10.1073/pnas.74.9.3942 PMC431793

[CIT0014] Carbone L , Harris RA, Gnerre S, Veeramah KR, Lorente-Galdos B, Huddleston J, Meyer TJ, Herrero J, Roos C, Aken B, et al. Gibbon genome and the fast karyotype evolution of small apes. Nature. 2014:513:195–201. 25209798 10.1038/nature13679 PMC4249732

[CIT0015] Chapuis M-P , Lecoq M, Michalakis Y, Loiseau A, Sword GA, Piry S, Estoup A. Do outbreaks affect genetic population structure? A worldwide survey in *Locusta migratoria*, a pest plagued by microsatellite null alleles. Mol Ecol. 2008:17:3640–3653. 18643881 10.1111/j.1365-294X.2008.03869.x

[CIT0016] Chapuis M-P , Loiseau A, Michalakis Y, Lecoq M, Franc A, Estoup A. Outbreaks, gene flow and effective population size in the migratory locust, *Locusta migratoria*: a regional-scale comparative survey. Mol Ecol. 2009:18:792–800. 19207256 10.1111/j.1365-294X.2008.04072.x

[CIT0017] Charlesworth D. Effects of inbreeding on the genetic diversity of populations. Philos Trans R Soc Lond B Biol Sci. 2003:358:1051–1070. 12831472 10.1098/rstb.2003.1296 PMC1693193

[CIT0018] Charlesworth D , Charlesworth B. Sex differences in fitness and selection for centric fusions between sex-chromosomes and autosomes. Genet Res. 1980:35:205–214. 6930353 10.1017/s0016672300014051

[CIT0019] Coyne JA. Correlation between heterozygosity and rate of chromosome evolution in animals. Am Nat. 1984:123:725–729.

[CIT0020] Coyne JA , Orr HA. Speciation. Sunderland (MA): Sinauer Associates; 2004.

[CIT0021] Crowson RA. The biology of the Coleoptera. London: Academic Press; 1981. p. xii+ 802.

[CIT0022] Drummond AJ , Rambaut A. BEAST: Bayesian evolutionary analysis by sampling trees. BMC Evol Biol. 2007:7:214. 17996036 10.1186/1471-2148-7-214 PMC2247476

[CIT0023] Duran DP , Gough HM. Validation of tiger beetles as distinct family (Coleoptera: Cicindelidae), review and reclassification of tribal relationships. *Syst Entomol*. 2020:45:723–729.

[CIT0024] Emerson BC , Oromí P, Hewitt GM. MtDNA phylogeography and recent intra-island diversification among Canary Island Calathus beetles. Mol Phylogenet Evol. 1999:13:149–158. 10508548 10.1006/mpev.1999.0644

[CIT0025] Felsenstein J. The evolutionary advantage of recombination. Genetics. 1974:78:737–756. 4448362 10.1093/genetics/78.2.737 PMC1213231

[CIT0026] Fisher RA. The genetical theory of natural selection. New York: Dover Publications; 1958.

[CIT0027] FitzJohn RG. Diversitree: comparative phylogenetic analyses of diversification in R. Methods Ecol Evol. 2012:3:1084–1092.

[CIT0028] Gohli J , Kirkendall LR, Smith SM, Cognato AI, Hulcr J, Jordal BH. Biological factors contributing to bark and ambrosia beetle species diversification. Evolution. 2017:71:1258–1272. 28257556 10.1111/evo.13219

[CIT0029] Gough HM , Allen JM, Toussaint EFA, Storer CG, Kawahara AY. Transcriptomics illuminate the phylogenetic backbone of tiger beetles. Biol J Linn Soc. 2020:129:740–751.

[CIT0030] Gough HM , Duran DP, Kawahara AY, Toussaint EFA. A comprehensive molecular phylogeny of tiger beetles (Coleoptera, Carabidae, Cicindelinae). Syst Entomol. 2019:44:305–321.

[CIT0031] Grant V. Plant speciation. New York (NY): Columbia University Press; 1981.

[CIT0032] Grégoire JC. In: Berryman A, editor. The greater European spruce beetle. Dynamics of Forest Insect Populations. New York: Plenum; 1988. p. 455–478.

[CIT0033] Guerrero RF , Kirkpatrick M. Local adaptation and the evolution of chromosome fusions. Evolution. 2014:68:2747–2756. 24964074 10.1111/evo.12481

[CIT0034] Handlirsch A. Die Fossilen Insekten und die Phylogenie der Rezenten Formen, parts I–IV. Ein Handbuch fur Palaontologen und Zoologen. Leipzig: Verlag Von Wilhelm Englelmann; 1906.

[CIT0035] Hill WG , Robertson A. The effect of linkage on limits to artificial selection. Genet Res. 1966:8:269–294. 5980116

[CIT0036] Hopkins DM , Matthews JV, Wolfe JA, Silberman ML. A Pliocene flora and insect fauna from the Bering Strait region. Palaeogeogr Palaeoclimatol Palaeoecol. 1971:9:211–231.

[CIT0037] Imai HT , Maruyama T, Crozier RH. Rates of mammalian karyotype evolution by the karyograph method. Am Nat. 1983:121:477–488.

[CIT0038] Kandul NP , Lukhtanov VA, Pierce NE. Karyotypic diversity and speciation in *Agrodiaetus* butterflies. Evolution. 2007:61:546–559. 17348919 10.1111/j.1558-5646.2007.00046.x

[CIT0039] King M. Species evolution: the role of chromosome change. Cambridge: Cambridge University Press; 1995.

[CIT0040] Kirkendall LR. Ecology and evolution of biased sex ratios in bark and ambrosia beetles. In: Evolution and diversity of sex ratio in insects and mites. New York: Chapman and Hall; 1993.

[CIT0041] Kitano J , Ross JA, Mori S, Kume M, Jones FC, Chan YF, Absher DM, Grimwood J, Schmutz J, Myers RM, et al. A role for a neo-sex chromosome in stickleback speciation. Nature. 2009:461:1079–1083. 19783981 10.1038/nature08441 PMC2776091

[CIT0042] Kunegel-Lion M , Lewis MA. Mountain pine beetle outbreak duration and pine mortality depend on direct control effort. J Environ Manage. 2020:260:110167. 32090789 10.1016/j.jenvman.2020.110167

[CIT0043] Labandeira CC , Lepage BA, Johnson AH. A *Dendroctonus* bark engraving (Coleoptera: Scolytidae) from a middle Eocene Larix (Coniferales: Pinaceae): early or delayed colonization? Am J Bot. 2001:88:2026–2039. 21669635

[CIT0044] Lande R. Effective deme sizes during long-term evolution estimated from rates of chromosomal rearrangement. Evolution. 1979:33:234–251. 28568063 10.1111/j.1558-5646.1979.tb04678.x

[CIT0045] Lande R. The fixation of chromosomal rearrangements in a subdivided population with local extinction and colonization. Heredity. 1985:54(Pt 3):323–332. 4019220 10.1038/hdy.1985.43

[CIT0046] Larochelle A , Larivière M-C. A natural history of the ground-beetles (Coleoptera: Carabidae) of America North of Mexico. London: Coronet Books Incorporated; 2003.

[CIT0047] Larson A , Prager EM, Wilson AC. Chromosomal evolution, speciation and morphological change in vertebrates: the role of social behaviour. In: Bennett MD, Gropp A, Wolf U, editors. Chromosomes Today: Volume 8 Proceedings of the Eighth International Chromosome Conference held in Lübeck, West Germany, 21–24 September 1983. Dordrecht: Springer Netherlands; 1984. p. 215–228.

[CIT0048] Lewis H. Speciation in flowering plants. *Science*. 1966:152:167–172. 17741624 10.1126/science.152.3719.167

[CIT0049] Lewontin RC. The effect of genetic linkage on the mean fitness of a population. Proc Natl Acad Sci USA. 1971:68:984–986. 5280533 10.1073/pnas.68.5.984 PMC389096

[CIT0050] Li S , Jovelin R, Yoshiga T, Tanaka R, Cutter AD. Specialist versus generalist life histories and nucleotide diversity in *Caenorhabditis* nematodes. Proc Biol Sci. 2014:281:20132858. 24403340 10.1098/rspb.2013.2858 PMC3896024

[CIT0051] Li Z , Tiley GP, Galuska SR, Reardon CR, Kidder TI, Rundell RJ, Barker MS. Multiple large-scale gene and genome duplications during the evolution of hexapods. Proc Natl Acad Sci USA. 2018:115:4713–4718. 29674453 10.1073/pnas.1710791115 PMC5939055

[CIT0052] Martynov AV. Ob iskopayemykh nasekomykh tretichnykh otlozheniy Ashutasa, Zaisanskogo uyezda [Fossil insects from Tertiary deposits in Ashutas, Saisan District]. Trudy Geol Muz Akad Nauk SSSR. 1929:5:173–216.

[CIT0053] McKenna DD , Farrell BD. Beetles (Coleoptera). In: The timetree of life. Oxford: Oxford University Press; 2009.

[CIT0054] Muller HJ. Some genetic aspects of sex. Am Nat. 1932:66:118–138.

[CIT0055] Muller HJ. The relation of recombination to mutational advance. Mutat Res. 1964:106:2–9. 14195748 10.1016/0027-5107(64)90047-8

[CIT0056] Nei M. Modification of linkage intensity by natural selection. Genetics. 1967:57:625–641. 5583732 10.1093/genetics/57.3.625 PMC1211753

[CIT0057] Olmo E. Rate of chromosome changes and speciation in reptiles. Genetica. 2005:125:185–203. 16247691 10.1007/s10709-005-8008-2

[CIT0058] Otto SP , Payseur BA. Crossover interference: shedding light on the evolution of recombination. Annu Rev Genet. 2019:53:19–44. 31430178 10.1146/annurev-genet-040119-093957 PMC8715713

[CIT0059] Petitpierre E. Why beetles have strikingly different rates of chromosomal evolution. Elytron. 1987:1:25–32.

[CIT0060] R Core Team. R: a language and environment for statistical computing. Vienna (Austria): R Foundation for Statistical Computing; 2021. https://www.R-project.org/

[CIT0061] Ratomponirina C. Synaptonemal complexes in Robertsonian translocation heterozygous in lemurs. In: Kew Chromosomes. London: HMSO Publ. Centre; 1988.

[CIT0062] Rieseberg LH. Chromosomal rearrangements and speciation. Trends Ecol Evol. 2001:16:351–358. 11403867 10.1016/s0169-5347(01)02187-5

[CIT0063] Roberts PA. A positive correlation between crossing over within heterozygous pericentric inversions and reduced egg hatch of *Drosophila* females. Genetics. 1967:56:179–187. 6040490 10.1093/genetics/56.1.179 PMC1211488

[CIT0064] Roberts PA. Screening for x-ray-induced crossover suppressors in *Drosophila melanogaster*: prevalence and effectiveness of translocations. Genetics. 1970:65:429–448. 5519644 10.1093/genetics/65.3.429 PMC1212456

[CIT0065] Ross L , Blackmon H, Lorite P, Gokhman VE, Hardy NB. Recombination, chromosome number and eusociality in the Hymenoptera. J Evol Biol. 2015:28:105–116. 25382409 10.1111/jeb.12543 PMC4328152

[CIT0066] Ruckman SN , Jonika MM, Casola C, Blackmon H. Chromosome number evolves at equal rates in holocentric and monocentric clades. PLoS Genet. 2020:16:e1009076. 33048946 10.1371/journal.pgen.1009076 PMC7584213

[CIT0067] Sherman PW. Insect chromosome numbers and eusociality. Am Nat. 1979:113:925–935.

[CIT0068] Suchard MA , Rambaut A. Many-core algorithms for statistical phylogenetics. Bioinformatics. 2009:25:1370–1376. 19369496 10.1093/bioinformatics/btp244 PMC2682525

[CIT0069] Sylvester T , Hjelmen CE, Hanrahan SJ, Lenhart PA, Johnston JS, Blackmon H. Lineage-specific patterns of chromosome evolution are the rule not the exception in Polyneoptera insects. Proc Biol Sci. 2020:287:20201388. 32993470 10.1098/rspb.2020.1388 PMC7542826

[CIT0070] Tarpy DR. Genetic diversity within honeybee colonies prevents severe infections and promotes colony growth. Proc Biol Sci. 2003:270:99–103. 12596763 10.1098/rspb.2002.2199 PMC1691209

[CIT0071] Templeton AR. Chromosome number, quantitative genetics and eusociality. Am Nat. 1979:113:937–941.

[CIT0072] Templeton AR. Mechanisms of speciation—a population genetic approach. Annu Rev Ecol Syst. 1981:12:23–48.

[CIT0073] White MJD. Modes of speciation. San Francisco: W.H. Freeman; 1978.

[CIT0074] Wickham HF. New Miocene Coleoptera from Florissant. Bull Mus Comp Zool. 1914:53:423–494.

[CIT0075] Wilson AC , Bush GL, Case SM, King MC. Social structuring of mammalian populations and rate of chromosomal evolution. Proc Natl Acad Sci USA. 1975:72:5061–5065. 1061091 10.1073/pnas.72.12.5061 PMC388875

[CIT0076] Wright S. On the probability of fixation of reciprocal translocations. Am Nat. 1941:75:513–522.

[CIT0077] Wright S. Isolation by distance under diverse systems of mating. Genetics. 1946:31:39–59. 21009706 10.1093/genetics/31.1.39 PMC1209315

